# Rotator Cuff Arthropathy: A Comprehensive Review

**DOI:** 10.1016/j.jhsg.2023.12.014

**Published:** 2024-02-29

**Authors:** Alexis L. Clifford, Eoghan Hurley, Oke Anakwenze, Christopher S. Klifto

**Affiliations:** ∗ University of Florida, College of Medicine, Gainesville, FL; † Department of Orthopaedic Surgery, Duke University, Durham, NC

**Keywords:** Arthritis, Arthroplasty, Arthroscopy, Cuff tear arthropathy, Massive rotator cuff tear, Rotator cuff arthropathy, Reverse total shoulder arthroplasty, Shoulder

## Abstract

Rotator cuff arthropathy is a spectrum of disease states secondary to full-thickness cuff tears classified by rotator cuff insufficiency and degenerative disease within the shoulder joint. Diagnosis can be made through standard physical exam and radiographic films demonstrating varying levels of weakness, along with acetabularization, femoralization, and superior migration of the humeral head. Severity of disease is classified through both the Hamada and Seebauer grading systems, which are used clinically to determine the appropriate treatment algorithm. Treatment exists along the spectrum from conservative therapy with physical therapy to a definitive treatment with total joint replacement. Depending on a patient’s progression and other comorbidities, arthroscopic treatments may additionally be used in specific circumstances as joint-sparing techniques. In recent years, reverse total shoulder arthroplasty has produced increasingly favorable outcomes with improvements in pain and function while simultaneously diminishing complication rates, making it generally accepted as standard of care. This disease limits quality of life for a large population of patients and efforts toward optimization of the treatment regimen is critical. This review provides an overview on the diagnostic criteria, classification, pathoanatomic changes, biomechanics, treatment options, outcomes, and complications of rotator cuff arthropathy.

## Rotator Cuff Arthropathy

Rotator cuff arthropathy (RCA) was described by Charles Neer in 1977 as a set of pathoanatomical findings secondary to chronic full-thickness rotator cuff tears. Neer postulated that erosion of the greater tuberosity of the humerus and restructuring of the coracoacromial arch allowed space for the proximal aspect of the humerus, creating the unique appearance of the joint within this subset of patients.^1^ Despite his inability to discern the pathogenesis, he hypothesized that 4% of patients with massive tears progress to RCA, a process that remains poorly understood.^1^^,^^2^

Recent diagnostic criteria state that RCA encapsulates the spectrum of pathologies defined by the presence of rotator cuff insufficiency, degenerative changes of the glenohumeral joint, and superior migration of the humeral head. Historically, nonuniform poorly defined treatment algorithms have produced mediocre outcomes for patients.^2^ Currently, the treatment algorithm begins conservatively with physical therapy, with those who fail commonly receiving reverse shoulder arthroplasty (RTSA) as definitive treatment. Despite the many surgical advances over the last decade, there remains room for improved understanding of how to achieve optimal outcomes for patients with RCA. This review is intended to provide an updated overview of the diagnosis, classification, pathoanatomy, biomechanics, treatment, outcomes, and complications of rotator cuff arthropathy.

## Diagnosis

The classic patient with RCA presents with progressive pain and limitation of activity, commonly in their dominant extremity.^3^ Although this may occur in patients of varying age groups, the majority of the patients are in their seventh decade or older.^3^ Hallmark findings of loss of motion coupled with significant weakness are easily attributable to damage of rotator cuff tendons; however, given that RCA encompasses a spectrum of disease states, it is better identified through a thorough physical exam and radiologic assessment.

Physical examination classically demonstrates weakness in supraspinatus and infraspinatus musculature with subcutaneous effusion, secondary to increased fluid pressure on the subacromial bursa, also occasionally seen.^2^ Special tests are used to further characterize strength of rotator cuff musculature, such as the lift-off test for subscapularis isolation and the Hornblower’s test for involvement of the teres minor.^2^ Passive and active range of motion (ROM) of the glenohumeral joint are commonly diminished because of weakness, pain, and/or stiffness. Specifically, presence or absence of an intact subscapularis may impact ROM due to changes within the force coupling and the anterior and posterior-superior rotator cuff muscles.^4^ This diminished ROM exists on a spectrum. Some develop compensatory strength in the deltoid, whereas others progress to pseudoparalysis in abduction and forward flexion.^2^ Anterosuperior escape of the humeral head may also be identified on exam with increased severity of disease.

Radiographic changes may additionally aid in diagnosis. The initial radiographic change seen for the acromion is sclerosis of the inferior aspect referred to as a sourcil sign. This is followed by acetabularization or thinning of the coracoacromial arch with destruction of the superior glenoid.^2^^,^^3^ Additionally, femoralization, defined as erosion of the greater tuberosity of the humerus, secondary to contact with the acromion, may be noted. Progressive superior migration of the humeral head seen as narrowing of the acromio-humeral distance, may also assist in identifying this pathology radiographically.^3^ Lastly, peripheral osteophyte formation is occasionally seen.^3^ Although plain radiographic film is typically sufficient with physical exam to diagnose RCA, computed tomography and magnetic resonance imaging may be beneficial for surgical planning to evaluate abnormal patterns of wear on the glenoid, and determine the integrity of specific tendons and quality of bone stock.^3^

## Classification

Two classification schemes exist for RCA: the Seebauer system and the Hamada system. The Seebauer system (Table 1) uses biomechanical metrics to delineate four grades of RCA based on the degree of superior migration from the center of rotation and the amount of instability around this point. Hamada’s system (Table 2) provides a mechanistic understanding of the radiographic changes seen in massive rotator cuff tears. This system is based upon the notion that all massive tears temporally progress to RCA through identifiable radiographic features. Grades of 1 through 5 are assigned based upon this progression in the size of the acromiohumeral interval and glenohumeral joint space.^5^ Both classification systems are useful clinically, providing similar inter- and intraobserver reliability and allowing communication regarding the severity of disease to dictate the type of treatment chosen by physicians.

**Table 1:** The Seebauer System

Seebauer System	
**Type I**	**Centered**
**Ia**	Dynamic joint stability + minimal superior migration
**Ib**	Compromised stability + minimal superior migration
**Type II**	**Decentered**
**IIa**	Insufficient stabilization + superior translation
**IIB**	Absent stabilization + anteriosuperior escape

**Table 2:** The Hamada System

Hamada System	
**Grade 1**	Acromiohumeral interval ≥6 mm
**Grade 2**	Acromiohumeral interval ≤5 mm
**Grade 3**	Acromiohumeral interval ≤5 mm + acetabularization
**Grade 4a**	Glenohumeral join narrowing without acetabularization
**Grade 4b**	Glenohumeral join narrowing with acetabularization
**Grade 5**	Bony destruction of the humeral head + collapse

## Pathoanatomy

RCA was first described as severe disorganization of the glenohumeral joint and collapse of the humeral head following massive rotator cuff tears.^1^ Erosion of the glenoid, acromion, clavicle, and acromioclavicular joint were additionally noted at varying degrees. Evidence of repair and collapse of the subchondral bone, hypervascular oestoporotic spongiosa, osteoarthritis at points of fixed contact, and atrophic cartiladge covering the humeral head were also correlated with this pathology.^1^ Now, the two key pathoanatomic sequela of RCA are femoralization and acetabularization. Femoralization is erosion of the glenoid and coracoid leaving the humerus rounded off and diminishing coverage from the rotator cuff, thus resembling the femoral head.^1^ Acetabularization is thinning of the coracoacromial arch with destruction of the superior glenoid. This is defined clinically as acetabular arthritis and radiographically by a concave deformity of the acetabular surface or coracoacromial arch.^5^ This concavity is classically accompanied by recession of the greater tuberosity and occasionally by excessive spur formation across the coracoacromial ligament.^5^

The two broad underlying theories of arthritic development are nutritional and mechanical. Nutritional theory suggests that the articular cartilage degradation is due to escape of nourishing factors secondary to the tear. This produces a reduction in pressure from joint fluid and thus a lack of sufficient nutrients to support the articular cartilage.^3^ The mechanical theory however suggests that deterioration of this articular cartilage is a product of abnormal physical stresses from the upward migration of the humeral head.^3^ This migration causes impingement and erosion with repetitive trauma of the articular surface leading to destruction of the cartilage. Others have suggested that the underlying pathophysiology is likely a combination; the humeral head impact produces cartilage fragmentation and debris, which incite an enzymatic response further damaging the cartilage.^3^

## Biomechanics

The healthy glenohumeral joint relies heavily on the rotator cuff musculature for both stabilization and ROM. To foster stability in the absence of osseous restraints, the rotator cuff maintains a centralized position for the humeral head within the glenoid fossa.^2^ For this stability to be attained, the forces exerted on the healthy joint must be balanced. When considering the vertical axis, the rotator cuff provides a net inferior force and a compressive vector, and the deltoid muscle produces a superior force within the shoulder joint. Through compression of the convex humeral head into the concave surface of the glenoid and labrum, the cuff therefore allows for concentric rotation of the head. These coupled forces are referred to as concavity-compression, and when disrupted, lead to imbalance across the joint.^2^^,^^6^ Maintenance of horizonal balance across the joint is additionally critical. As the only anterior cuff muscle, the subscapularis is responsible for resisting anterior-inferior translation and is balanced by the infraspinatus and teres minor musculature posteriorly to provide a balanced set of forces.^4^ Massive rotator cuff tears and degeneration inherently uncouple these sets of forces. This loss of integrity ultimately culminates in upward migration of the humeral head alongside potential bony erosion of the superior glenoid and acromion and an unstable fulcrum of motion.^2^^,^^7^

## Treatment

Along the spectrum of disease there exist many different treatment options categorized as conservative treatment, arthroscopic interventions, and arthroplasty.

### Conservative treatment

Currently, the first-line nonsurgical treatments include activity modification, nonsteroidal anti-inflammatory drugs, subacromial corticosteroid injection and physical therapy with scapular and rotator cuff strengthening.^2^^,^^8^ Although intraarticular corticosteroid injections may initially be effective in reducing pain, repeated use is discouraged due to diminishing utility.^2^ Physical therapy however has proven effective in the treatment of RCA along the full spectrum of disease.^9^ Therapy is typically aimed at strengthening the anterior deltoid with additional focus on pectoralis major and latissimus dorsi musculature to improve glenohumeral kinematics.^10^ Although this treatment modality has demonstrated clinical successes for many, failure has been shown to be correlated to patients having at least three tendon tears or anterior cuff involvement.^9^ In these circumstances, surgical intervention may be required secondary to humeral head decentering and loss of force coupling.^9^^,^^10^

### Arthroscopic treatment

A wide variety of arthroscopic treatments are utilized as joint-sparing surgical interventions for patients with RCA. The goal of arthroscopic rotator cuff repair is anatomic restoration, and the procedure is mostly performed in the absence of arthritis to prevent further degeneration and progression of RCA. Unfortunately, many tears are either not amenable to repair or are at high risk of retear, making use of this procedure complex and infrequent.^10^ Arthroscopic debridement or partial repairs are also used as prevention in the earlier radiographic stages of RCA however there remains some controversy on indications and efficacy.^8^^,^^10^ At large, these reparative techniques may produce promising results for less severe cases of RCA, however, further research is needed to better identify which patients may benefit and how to optimize their outcomes.

Arthroscopic superior capsular reconstruction (SCR) with a tensor fascia lata autograft is another technique used. SCR aims to promote deltoid function by preventing superior migration of the humeral head, relieve pain, and restore motion of the glenohumeral joint.^10^^,^^11^ Indications for use of this procedure have been debated. The initial indication for use was patients with good passive ROM, intact infraspinatus and supraspinatus, or poor candidates for RTSA; however, recent data have demonstrated this procedure may produce similar outcomes in patients along the spectrum of disease when glenohumeral joint space is maintained.^10^, ^11^^,^^11^ These data demonstrate durability and success with production and maintenance of significant improvements through 2 years after surgery.^11^

Patients with irreparable posterior-superior rotator cuff tears may be treated with lower trapezius transfer to improve external rotation strength. This has demonstrated improved outcomes in patients with Hamada grade 1 and 2 RCA but has been correlated to poorer outcomes in patients with more severe disease. Similarly, patients with irreparable anterosuperior rotator cuff tear may benefit from combined latissimus dorsi and teres major tendon transfer. This procedure aims to restore joint kinematics and has been reported to provide promising clinical and radiologic outcomes in this population.^12^ This combinatory procedure provides a thicker more effective transfer capable of producing dynamic shoulder stability without rupture. Moreover, this technique significantly decreased contact pressure and did not culminate in progression of RCA within 2 years of follow-up.^12^ These results place combined tendon transfer as a feasible joint sparing treatment methodology for this subset of patients. In general however, significant joint arthropathy limits the utility of any of these arthroscopic techniques suggesting the need for arthroplasty.

### Arthroplasty: reverse total shoulder arthroplasty (RTSA)

Two arthroplasty procedures have historically been used for treatment of RCA, including RTSA and cuff tear arthropathy hemiarthroplasty (CTAH); however, RTSA is currently standard of care.^2^^,^^10^ The biomechanical design of the RTSA implant has promoted its use for RCA as it empowers elevation of the shoulder without relying on the rotator cuff. The joint center of rotation is medialized and distalized, altering the mechanics of the shoulder. As such, shoulder elevation remains possible through the deltoid force vector, which is not typically damaged at presentation with this pathology.^10^ Additionally, the reversed nature produces a stable and semiconstrained environment, allowing the prosthesis to stand alone without the superior restraint classically provided by the coracoacromial arch. As this procedure has become optimized, less medialization of the glenoid prosthesis has been implemented, allowing for an increase in ROM through more uniform distribution of force across the bone-baseplate interface.^10^

Data suggest that most patients are apt to benefit from RTSA along the spectrum of disease. Historically, it was thought that younger patients (<65 years) should avoid RTSA in favor of CTAH to spare the glenoid and decrease their risk of complications and revision. Recent data however suggest that RTSA provides younger patients who are experiencing loss of function with increased satisfaction without an increased likelihood of implant failure or revision, compared to those over 65 years.^2^^,^^13^ Additionally, individuals with external or internal rotation pseudoparalysis benefit from RTSA in conjunction with tendon transfers to support remobilization. Patients with external rotation paralysis may benefit from latissimus dorsi transfer, whereas those with internal rotation deficiency or subscapularis insufficiency may benefit from pectoralis transfer. Additionally, there are some data to suggest that a lateralized glenosphere may allow reversal of this external rotation pseudoparalysis as well. These specific patient populations require excess surgical manipulation to optimize their functional outcomes after surgery.

## Surgical Outcomes

Outcomes from arthroscopic treatments for RCA are heterogenous. The use of debridement, partial repair and complete repair remain controversial. Regarding debriedment, some data demonstrate decreased pain and improved function or ROM scores wheras other depict no improvements.^8^, ^10^ It additionally remains unclear whether partial repair differs from either simple debreidment or complete repair with regards to functional outcomes and retear rates.^10^ SCR however has demonstrated success for patients in the early stages of disease progression. This procedure decreases contact pressure, cumulative deltoid forces, and superior migration, generating improved functional outcomes for patients.^14^ Despite this, it only partially restores the native load of the glenohumeral joint and as such produces a high graft failure rate.^14^ This raises concern regarding true joint-preservation ability as progression to RTSA may be imminent.^14^

At large, outcomes after RTSA demonstrate clinically significant improvements in both function and pain, reinforcing this procedure as the surgical standard of care.^10^^,^^15^^,^^16^ Specifically, patients significantly improve active ROM in flexion, abduction, and external rotation as well as increases in both the constant and American Shoulder and Elbow Surgeons scores greater than the minimal clinically important difference.^15^ Interestingly, patient-reported satisfaction after RTSA was correlated with severity of disease, with those with more severe disease corresponding to higher satisfaction after surgery.^16^ Although some studies have demonstrated improvements along the spectrum of disease, others have identified specific subpopulations that have been associated with inferior outcomes, including patients with less severe disease noted as an absence of pseudoparalysis, higher preoperative function, and younger age.^16^ Additionally, history of failed cuff repair negatively impacts the results of subsequent RTSA, producing inferior results.^17^ Recent data on outcomes after combination of RTSA with anterior latissimus dorsi and teres major tendon transfer have demonstrated improved clinical and functional outcomes regarding internal rotation compared to RTSA alone.^18^ These data suggest use of tendon transfer in the subset of patients apt to benefit. At large, RTSA generates meaningful improvements for patients with RCA.

## Complications

Complication and revision rates after RTSA have dramatically improved over the last decade. A systematic review from 2005 to 2020 noted the overall complication rate for RTSA was 9.4%, with an overall pooled revision rate of 2.6%, down from historic figures of 24% and 10.1%, respectively.^15^ This study further subdivided the complications into major medical (0.07%) shoulder- or surgical related (5.3%) and infections (1.2%) highlighting the most commonly cited surgical complications of periprosthetic fractures, dislocation or instability, and glenoid component loosening.^15^ Amongst these complications, the most common to require revision is glenoid component loosening or failure; however, all contribute to the overall burden of revision.^10^ Comparison to earlier systematic review highlights the impact of changes to these prostheses lateralizing the center of rotation and utilizing a more anatomic neck-shaft angle. While these changes diminished the incidence of common complications such as scapular notching and improved overall impingement-free ROM, these design changes may have inadvertently resulted in the increased rates of postoperative acromial and scapular spine fractures noted.^15^ Advancements in the techniques and implants have led to decreases in complication and revision rates at large throughout recent years however the potential for further optimization remains.

## Postoperative Rehabilitation

Postoperative rehabilitation for RCA varies greatly. The protocol hinges on both the surgical procedure performed and the individual patient’s status and motivation to improve. Heterogeneity specifically exists among recommendations regarding use of sling, when to begin active and passive ROM, what resistance exercises to do, and what precautions to take.^19^ A systematic review on rehabilitation after RTSA demonstrated time frames ranging from 12 weeks to 6 months.^19^ Despite this variation, all included studies suggested initiation of passive ROM and resistance exercise by week 6 along with full passive and active ROM by week 12.^19^ A specific question of interest was followed up with a randomized control trial on immediate rehabilitation versus delayed with immobilization after surgery.^20^ Results demonstrated that both time courses produced statistically and clinically significant improvements in American Shoulder and Elbow Surgeons scores and patient recorded outcome measurements within 6 weeks.^20^ The study additionally demonstrated no significant difference in dislocation rates or opioid use and while a statistically significant difference in pain existed favoring delayed therapy, this did not translate to a clinically significant difference.^20^ This study supports the safety of early initiation of therapy to avoid complications associated with prolonged immobilizations; however, it does not provide data to support one methodology over the other. Further studies on the timing and method of postoperative rehabilitation are critical to optimize patient recovery.

## Conclusion

The ability to treat RCA has become more refined over recent decades with improved characterization of the pathophysiology and development of this disease state. Advancements over the last several decades in the technology and techniques available have progressively improved outcomes and reduced complication rates for patients with both mild and severe disease. Further research describing the benefits associated with less invasive treatments in specific patient populations will aid in the production of superior outcomes. Optimizing the ability to diminish pain and improve function for this patient population will greatly reduce the burden of disease and allow surgeons to provide high-value care moving forward.

## Conflicts of Interest

No benefits in any form have been received or will be received related directly to this article.
